# Functions of Protosilencers in the Formation and Maintenance of Heterochromatin in *Saccharomyces cerevisiae*

**DOI:** 10.1371/journal.pone.0037092

**Published:** 2012-05-17

**Authors:** Xinmin Zhang, Qun Yu, Lars Olsen, Xin Bi

**Affiliations:** 1 Department of Biology, University of Rochester, Rochester, New York, United States of America; 2 School of Pharmaceutical Sciences, Jilin University, Changchun, Jilin, People's Republic of China; Duke University, United States of America

## Abstract

In *Saccharomyces cerevisiae*, transcriptionally silent heterochromatin at *HML* and *HMR* loci is established by silencers that recruit SIR complex and promote its propagation along chromatin. Silencers consist of various combinations of two or three binding sites for origin recognition complex (ORC), Abf1 and Rap1. A single ORC, Abf1 or Rap1 site cannot promote silencing, but can enhance silencing by a distant silencer, and is called a protosilencer. The mechanism of protosilencer function is not known. We examine the functions of ORC, Abf1 and Rap1 sites as components of the *HMR-E* silencer, and as protosilencers. We find that the Rap1 site makes a larger and unique contribution to *HMR-E* function compared to ORC and Abf1 sites. On the other hand, Rap1 site does not act as a protosilencer to assist *HML-E* silencer in forming heterochromatin, whereas ORC and Abf1 sites do. Therefore, different mechanisms may be involved in the roles of Rap1 site as a component of *HMR-E* and as a protosilencer. Heterochromatin formed by ORC or Abf1 site in collaboration with *HML-E* is not as stable as that formed by *HMR-E* and *HML-E*, but increasing the copy number of Abf1 site enhances heterochromatin stability. ORC and Abf1 sites acting as protosilencers do not modulate chromatin structure in the absence of SIR complex, which argues against the hypothesis that protosilencers serve to create a chromatin structure favorable for SIR complex propagation. We also investigate the function of *ARS1* containing an ORC site and an Abf1 site as a protosilencer. We find that *ARS1* inserted at *HML* enhances heterochromatin stability, and promotes *de novo* formation of a chromatin structure that partially resembles heterochromatin in an S phase dependent manner. Taken together, our results indicate that protosilencers aid in the formation and maintenance of heterochromatin structure.

## Introduction

Transcriptional silencing in *Saccharomyces cerevisiae* is a form of region specific gene repression that exists at the *HML* and *HMR* loci and subtelomeric regions [1]. It is mediated by heterochromatin established *via* the association of the SIR silencing complex consisting of Sir2 through Sir4 with nucleosomes. Heterochromatin is a stable but dynamic structure [2]. It is relatively refractory to DNA modifying and repair enzymes as well as endonucleases [3]–[7]. On the other hand, it is permissive to homologous or site-directed recombination as well as transposon integration [8]–[10]. Nucleosomes in heterochromatin are generally regularly ordered and are hypoacetylated compared to those in euchromatin [11]–[14]. As a reflection of the special structure of heterochromatin, DNA in heterochromatin is more negatively supercoiled than that in euchromatin [8], [9].

Formation of heterochromatin at the cryptic mating loci *HML* and *HMR* is promoted by small cis-acting elements called the *E* and *I* silencers flanking these loci [1]. Silencers each contain two or three recognition sites for ORC (origin recognition complex for DNA replication), Rap1 and Abf1. These silencer-binding proteins can interact with the Sir3 and Sir4 proteins in the SIR complex on their own or through Sir1 thereby recruiting them to the silencers. Sir2 is a histone deacetylase that is responsible for hypoacetylation of heterochromatin [15]. The SIR complex also binds to nucleosomes with a strong preference for unacetylated ones [16]–[21]. In addition, SIR complex self interacts and is able to form multisubunit chains. The current model for the *de novo* formation of heterochromatin proposes that SIR complexes recruited to a silencer deacetylate histones in adjacent nucleosomes. The newly deacetylated nucleosomes then bind additional SIR complexes. Through repeated cycles of histone deacetylation and SIR complex recruitment, SIR complexes are believed to spread along a continuous array of nucleosomes during which the primary chromatin structure pertaining to the distribution of nucleosomes along DNA is altered [1], [11], [12], [22]. The spreading model for heterochromatin formation is supported by our finding that nucleosome-excluding structures can block the propagation of heterochromatin [23].

The function of a silencer is affected by other silencers or protosilencers present in its surroundings. Protosilencers are DNA elements that can enhance the activity of a silencer at a distance without the ability to act as *bona fide* silencers on their own [24]. Single recognition sites for silencer-binding proteins have protosilencer activities [2], [24]–[27]. Silencers and protosilencers are collectively referred to as silencing elements. There have been many documented examples of two silencing elements cooperating to promote stronger silencing, but the underlying mechanisms have not been resolved [2], [24]–[27]. The fact that two silencers separated by up to several kb are able to cooperate to silence a reporter gene located between them can be explained by assuming that convergent spreading of Sir proteins emanating from the silencers is additive or synergistic, so that heterochromatin established between the silencers is stronger than that formed by either silencer alone [28]. However, this interpretation does not apply to silencer-protosilencer cooperation since a protosilencer is not able to initiate *de novo* silencing. It is possible that distant silencing elements cooperate by physically interacting with each other or with a common nuclear structure to create a stronger platform for recruiting Sir proteins [24], [26]. Alternatively, or in addition, because a protosilencer is actually a binding site for ORC, Abf1 or Rap1 that has the potential of positioning nucleosomes [23], [29]–[33], it is conceivable that a protosilencer modulates nucleosome positioning in the region between it and the silencer in a configuration that is more favorable for SIR complex spreading from the silencer [27]. However, definitive evidence for either hypothesis is lacking.

In this work, we examined the functions of the ORC, Rap1 and Abf1 binding sites as constituents of the *HMR-E* silencer, and as protosilencers. We found that the Rap1 site played a larger role than ORC or Abf1 site in *HMR-E* function, and imparted a unique property to the silencer. On the other hand, ORC and Abf1 sites aided in the formation of heterochromatin by the *HML-E* silencer, but Rap1 site did not. ORC and Abf1 sites acting as protosilencers did not affect local chromatin structure in the absence of SIR complex, which argues against the hypothesis that a protosilencer assists the formation of heterochromatin by creating a chromatin structure favorable for the spread of SIR complex. We also examined the function of *ARS1* containing an ORC site and an Abf1 site as a protosilencer. We found that *ARS1* inserted at *HML* significantly enhanced the stability of *HML* heterochromatin, and had the ability to promote *de novo* formation of a SIR-dependent chromatin conformation that partially resembled heterochromatin.

## Materials and Methods

### Plasmids

Plasmid pYZ167-I was made by replacing the EcoRI-HindIII fragment of pUC19 with an EcoRI-HindIII fragment corresponding to coordinates 290027 to 291756 of chromosome III that contains the *HMR-E* silencer (291245 to 291560). The *KanMX* cassette was inserted at the EcoRV site of pYZ167-I to make pQY298. pQY299 was derived from pQY298 by replacing the Abf1-binding site (BS) (5′-TCATAAAATACGAACG-3′) in *HMR-E* with an MfeI restriction site (CAATTG) *via* site-directed mutagenesis. pQY300 and 301 were similarly made by replacing the ORC-BS (TAAATATAAAA) and Rap1-BS (AAAACCCATCAACCT) in *HMR-E* with SpeI sites (ACTAGT). The genomic fragment HindIII-*HMR*-Hind III (289227–294210) from chromosome III was inserted into pUC12, making pUC-HMR. The MfeI-*HMR-I*-XhoI fragment of pUC-HMR was replaced by an MfeI-*HIS3*-XhoI fragment to make pQY321. Plasmid pQY226 was made by first replacing the AatII-BamHI fragment of pUC12 with the AatII-BamHI fragment of chromosome III (12139–16269) containing the *HML-I* silencer, and then replacing the HpaI-*HML-I*-HindIII fragment with HindIII-*HMR-E*-HindIII fragment, followed by inserting the *URA3* gene at the EcoRV site. The Rap1-BS and Abf1-BS in *HMR-E* in pQY226 were replaced by SpeI and MfeI sites, respectively, via site-directed mutagenesis to make plasmid pLO29. The ORC-BS and Rap1-BS in *HMR-E* in pQY226 were replaced by MfeI and SpeI sites, respectively, to make plasmid pLO28. The ORC-BS, Rap1-BS and Abf1-BS in *HMR-E* in pQY226 were replaced by KpnI, SpeI and MfeI sites, respectively, to make plasmid pLO30. The Rap1-BS from *HMR-E* was inserted at the MfeI site of pLO30 to make plasmid pLO40. The Abf1-BS from *HMR-E* was inserted at the SpeI site of pLO28 to make pLO33. Abf1-BS was inserted at the KpnI site of pLO33 to make pLO34. Plasmid pXB133-1 was made by inserting a BsrGI-*ARS1*-BsrGI sequence of chromosome IV (462460 - 262670) into plasmid pYXB5 [8]. Plasmid pUC26 was made by inserting the BamHI-*HML*-BamHI fragment (9666 to 16269 of chromosome III) into pUC12. Plasmid pYZ121 was made by replacing the HpaI-*HML-I*-HindIII fragment of pUC26 with HindIII-*HMR-E*-HindIII fragment, and inserting *URA3* gene at the BspHI site. The mutant alleles of HindIII-*HMR-E*-HindIII from plasmids pLO29, pLO28, pLO40, pLO30, pLO33 and pLO34 were used to replace the HindIII-*HMR-E*-HindIII fragment in pYZ121 to make pXZ31, pXZ33, pXZ34, pXZ35, pXZ37 and pXZ38, respectively.

### Yeast strains

Strains 1, 3, 5 and 7 (Table S1) were made by transforming strain CCFY101 to G418 (geneticin)-resistance with EcoRI and XbaI digested plasmids pQY298 through 301, respectively. The *SAS2* coding region in strains 1, 3, 5 and 7 was replaced by *NatMX* to make strains 2, 4, 6 and 8, respectively. Strains 1, 3, 5 and 7 were transformed to His^+^ by plasmid pQY321 digested with AatII and XbaI, making strains 9, 11, 13 and 15, respectively. Strains 10, 12, 14 and 16 were derived from 9, 11, 13 and 15, respectively, by replacing *SAS2* with *NatMX*. Strains 17, 18, 19, 20, 21, 22 and 23 were made by transforming strain YXB6 to Ura^+^ with BspHI and NgoMIV digested plasmids pQY226, pLO29, pLO28, pLO40, pLO30, pLO33 and pLO34, respectively. The *SIR3* gene in strains 17 through 21 were replaced with *KanMX*, making strains 17s through 21s, respectively. Strains 17n through 23n were made by transforming strain YXB6 to Ura^+^ with BlpI and NgoMIV digested plasmids pYZ121, pXZ31, pXZ33, pXZ34, pXZ35, pXZ37 and pXZ38, respectively. Strain 24 was made by transforming Y2047b to canavanine resistance with BamHI and NgoMIV digested plasmid pXB133-1. Strain 24s was derived from 24 by disrupting its *SIR3* gene with *URA3* as described [34]. Strain 25 was from E. Xu and J.R. Broach (Princeton University). Strain 26 was made by transforming YXB5s to G418 resistance with Tth111I digested plasmid pUC-SK [35]. Strain 27 was similarly derived from YXB125s.

### Analysis of the supercoiling of DNA circles from yeast

Yeast cells were grown in YPR medium (1% yeast extract, 2% bacto-peptone and 2% raffinose). When needed, galactose was added to YPR cultures at a concentration of 2%. α-factor, hydroxyurea (HU), and nocodazole were used at 10 µg/ml, 0.2 M and 20 µg/ml, respectively. Nucleic acids were isolated from yeast cultures using the glass bead method and fractionated on agarose gels in 0.5× TPE (45 mM Tris, 45 mM phosphate, 1 mM EDTA, pH 8.0) supplemented with chloroquine. DNA circles were detected by Southern blotting.

### Chromatin mapping by micrococcal nuclease (MNase) digestion and indirect end-labeling

This was done as described before [23], [36]. Briefly, about 2×10^8^ permeabilized spheroplasts prepared from log phase cells were treated with MNase at 15 and 30 units/ml, respectively, at 37°C for 4 minutes, and the DNA was isolated. DNA in each sample was then digested with SnaBI and EcoNI, and run on a 1.0% agarose gel. Relevant DNA fragments were visualized by using a specific probe after Southern blotting.

## Results

### The ORC-, Abf1- and Rap1-binding sites in the *HMR-E* silencer differentially contribute to its silencing function

**Figure 1:**
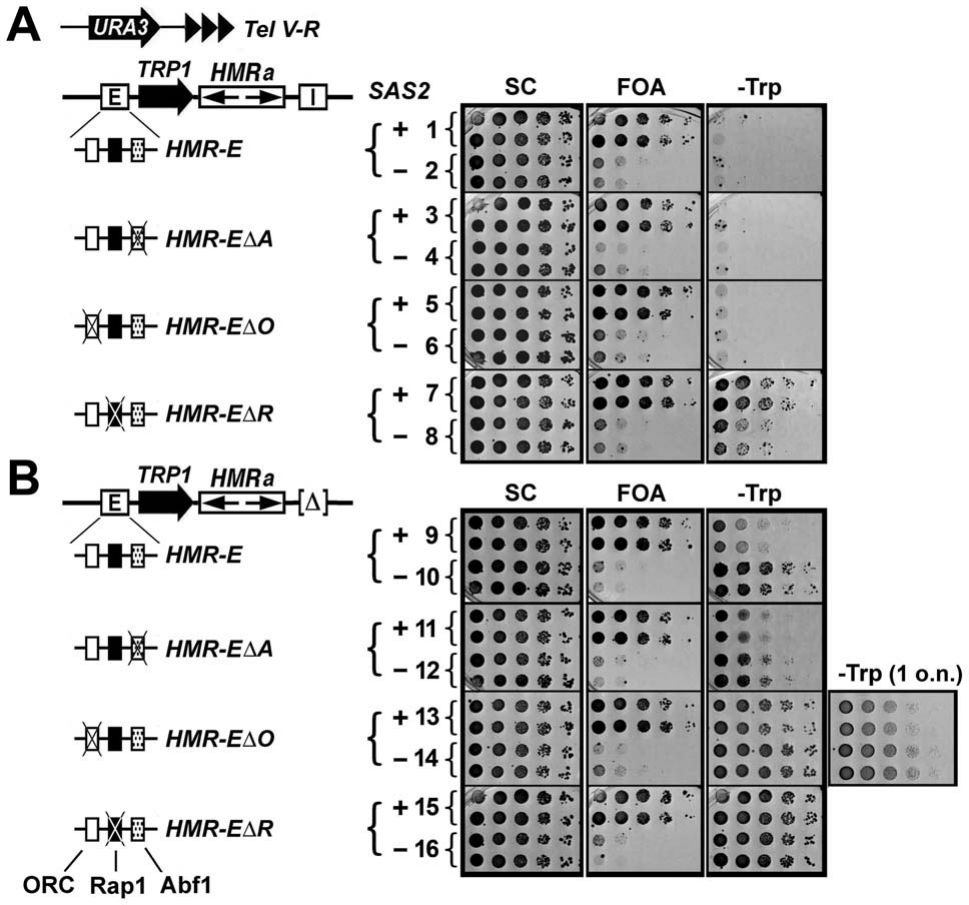
Effects of deleting the ORC-, Abf1- or Rap1-binding site from *HMR-E* on transcriptional silencing at *HMR*. (A) Effects of *HMR-E* mutations on *HMR*-silencing in the presence of *HMR-I* silencer. The silencing reporters *Tel V-R-URA3* and *HMR::TRP1* are illustrated at the top. The intact and mutant *HMR-E* silencers are shown on the left. Serial 10 fold dilutions of two independent clones of each of strains 1 through 8 were incubated at 30°C for two overnights on synthetic complete (SC), SC + 5-fluoroorotic acid (FOA), and SC lacking tryptophan (-Trp) media. The growth phenotypes are shown on the right. (B) Effects of *HMR-E* mutations on *HMR*-silencing in the absence of *HMR-I*. Growth phenotypes of strains 9 through 16 on SC, FOA and -Trp media are shown on the right. Note growth phenotypes of strains 13 and 14 on –Trp medium after one overnight (1 o.n.) incubation are also shown.

The *HMR-E* silencer is composed of one each of ORC-, Abf1- and Rap1-binding sites (abbreviated as -BSs hereafter). It was originally shown that deletion of any one of these sites did not affect the silencing of the resident *HMRa1* gene at *HMR*, whereas deletion of any two sites abolished *HMRa1* silencing [37]. This result suggests that ORC-, Abf1- and Rap1-BSs play similar and redundant roles in the function of *HMR-E*. Since the apparent efficiency of silencing by a silencer depends on the strength of the promoter of the reporter gene [38], the sensitivity/resolution of a silencing assay may depend on the reproter gene used. We attempted to further examine the contributions of the ORC-, Abf1- and Rap1-BSs to *HMR-E* function using an alternative reporter gene *TRP1* that is required for tryptophan biosynthesis. Strain 1 has its endogenous *TRP1* gene removed, and has *TRP1* with its own promoter inserted within *HMR* (Fig. 1A, left). It also has the *URA3* reporter gene inserted near the right telomere of chromosome V (*Tel V-R*) (Fig. 1A, left). Therefore, strain 1 allows for simultaneous examination of both *HMR* and telomere silencing. Silencing of *TRP1* was robust as cells failed to grow on medium lacking tryptophan (-Trp) (Fig. 1A, -Trp panel, 1). Deletion of ORC- or Abf1-BS from *HMR-E* had no effect on *TRP1* silencing (Fig. 1A, -Trp panel, 3 and 5), which is consistent with results from earlier studies on *HMRa1* silencing [37]. However, removal of Rap1-BS from *HMR-E* significantly reduced *TRP1* silencing (Fig. 1A, -Trp panel, compare 7 with 1). Therefore, using *TRP1* as a silencing reporter, we have revealed that Rap1-BS in *HMR-E* contributes more to *HMR-E* function than the ORC- or Abf1-BS. Removal of Rap1-BS prevents Rap1 from binding to *HMR-E*, which is likely the cause of the reduction in silencing. In the meantime, removal of Rap1-BS in strain 7 also decreases the distance between ORC- and Abf1-BSs in *HMR-E*, which may also affect the efficiency of silencing. *URA3* near *Tel V-R* was silenced, which was not affected by the mutations of the *HMR-E* silencer, as expected (Fig. 1A, robust growth of strains 1, 3, 5 and 7 on FOA medium; note cells expressing *URA3* are sensitive to killing by FOA, 5-fluoroorotic acid).

The *HMR-I* silencer plays an auxiliary role in *HMR* silencing [39]. The roles of the ORC- and Abf1-BSs in *HMR-E* function might be better revealed in the absence of *HMR-I*. Along this line, we deleted *HMR-I* from strains 1, 3, 5 and 7 to make strains 9, 11, 13 and 15, respectively (Fig. 1B, left). In the absence of *HMR-I*, *TRP1* silencing by *HMR-E* was moderately reduced (Fig. 1, -Trp panel, compare 9 with 1). *TRP1* silencing by *HMR-EΔA* (*HMR-E* lacking Abf1-BS) was slightly less efficient than that by *HMR-E* (Fig. 1B, -Trp panel, compare 11 with 9). Therefore, Abf1-BS was mostly dispensable for *TRP1* silencing even in the absence of *HMR-I*. On the other hand, deletion of ORC-BS markedly reduced *TRP1* silencing (Fig. 1B, -Trp panel, compare 13 with 9), and Rap1p-BS deletion eliminated *TRP1* silencing (Fig. 1B, -Trp panel, compare 15 with 9). Taken together, the above results demonstrate that Abf1-, ORC- and Rap1-BSs make increasingly larger contributions to silencing by the *HMR-E* silencer.

### The positive regulation of *HMR-E* by *SAS2* depends on the presence of the Rap1-BS, not ORC-BS or Abf1-BS of the silencer

*SAS2* encoding a histone H4 acetyltransferase is required for telomeric silencing and full silencing by the *HMR-E* silencer [40]–[43]. However, *SAS2* plays an inhibitory role in silencing by *HMR-E* with both its Rap1- and Abf1-BSs mutated [40], [44]. It is possible that the positive role of *SAS2* in *HMR-E* function depends on the presence of Rap1-BS and/or Abf1-BS in the silencer. We set out to determine whether it is Rap1- or Abf1-BS that is required for *SAS2* to positively regulate *HMR-E*. To this end, we deleted *SAS2* from strains 1, 3, 5, 7, 9, 11, 13 and 15, making strains 2, 4, 6, 8, 10, 12, 14 and 16, respectively (Fig. 1).

We showed that in the presence of *HMR-I*, *sas2Δ* significantly enhanced *TRP1* silencing by *HMR-EΔR* (Fig. 1A, note that growth of strain 8 on –Trp medium was significantly less robust than that of strain 7), suggesting that *SAS2* negatively regulates the function of *HMR-EΔR*. On the other hand, *TRP1* silencing by intact *HMR-E*, *HMR-EΔA* or *HMR-EΔO* was not affected by *sas2Δ* (Fig. 1A, -Trp, compare 2, 4 and 6 with 1, 3 and 5, respectively).

In the absence of *HMR-I*, *sas2Δ* markedly reduced *TRP1* silencing by *HMR-E* (Fig. 1B, -Trp, compare 10 with 9). *TRP1* silencing by *HMR-EΔA* or *HMR-EΔO* was also decreased by *sas2Δ*, albeit to lesser extents (Fig. 1B, note that growth of 12 and 14 was moderately more robust than 11 and 13, respectively, on –Trp medium). *HMR-EΔR* failed to silence *TRP1*, which was not affected by *sas2Δ* (Fig. 1B, -Trp, compare 16 with 15). Note, as expected, *sas2Δ* abolished the silencing of *URA3* near *Tel VR*, which is independent of the status of *TRP1* silencing at *HMR* (Fig. 1, FOA panel).

The above results suggest that *SAS2* positively regulates the function of *HMR-E*, as well as *HMR-EΔA* and *HMR-EΔO*, but negatively regulates *HMR-EΔR.* Therefore, the presence of Rap1-BS in *HMR-E* imparts a unique property to the silencer regarding regulation by *SAS2*.

### The ORC-, Abf1- and Rap1-BSs from the *HMR-E* silencer have distinct protosilencer activities

The fact that individually deleting the ORC-, Abf1- and Rap1-BSs from *HMR-E* reduces the silencing function of the silencer to different extents demonstrates that these elements do not contribute equally to *HMR-E* function (Fig. 1). It is not known whether the activities of these elements in the context of *HMR-E* silencer are related to their functions as protosilencers. To address this question, we set out to examine the ability of each element to facilitate the *HML-E* silencer in establishing heterochromatin. The structure of heterochromatin was examined by probing the topology of its DNA. This method is based on the fact that formation of a nucleosome constrains on average one negative supercoil on nucleosomal DNA, which is reduced by nucleosome acetylation, and hence the negative supercoiling of eukaryotic DNA in a locus is mainly determined by nucleosome density and conformation [45], [46]. Consistently, we and others have previously shown that DNA in heterochromatin at *HML* or *HMR* is characteristically more negatively supercoiled when the locus is silenced than when it is derepressed [8], [9].

**Figure 2:**
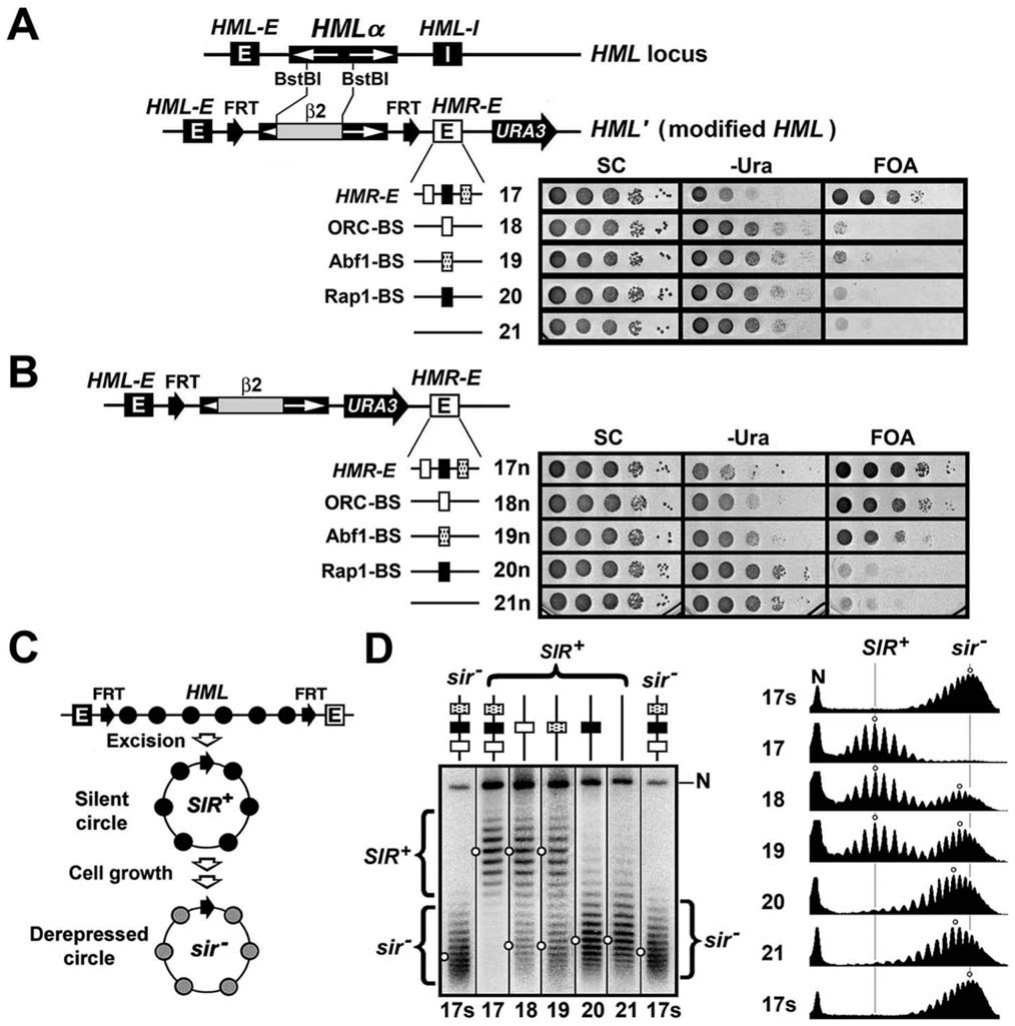
Cooperation of protosilencers ORC-BS, Abf1-BS and Rap1-BS with the *HML-E* silencer in forming heterochromatin at *HML*. (A) The schematics of the original *HML* locus and the modified *HML* locus (*HML′*) in strains 17 through 21 are shown on the left. In strains 17 through 21, the BstBI-BstBI fragment containing the divergent promoters and a portion of coding regions of the *α1* and *α2* genes at *HML* was replaced by a sequence designated β2 from the coding region of the *E. coli lacZ* gene, as has been described previously [8], and two FRTs in the same orientation were inserted at *HML*. The *HML-I* silencer was replaced by *HMR-E* (strain 17), ORC-BS (strain 18), Abf1-BS (strain 19), or Rap1-BS (strain 20). *HML-I* was replaced by *HMR-E* lacking all three binding sites in strain 21. Right, growth phenotypes of strains 17 through 21 on SC, -Ura and FOA media. (B) Left, schematics of *HML′* in strains 17n through 21n. Right, growth phenotypes of strains 17n through 21n. (C) Method for examining the structure and stability of *HML* heterochromatin. Top, *HML* locus excluding silencers is flanked by two FRTs. Recombination between the FRTs by Flp1 excises the *HML* circle without silencers. Heterochromatin on the circle is subject to disruption (changing to derepressed chromatin) during cell growth (cell cycle progression) [8]. Nucleosomes in heterochromatin and derepressed chromatins are marked by filled and shaded circles, respectively. (D) Analysis of *HML* DNA supercoiling. Cells of each strain grown in YPR to late log phase were treated with 2% galactose for 2.5 hr. Nucleic acids were isolated and fractionated in the presence of 26 µg/ml chloroquine. Topoisomers of *HML* circles from strains 17 and 17s were labeled *SIR* ^+^ and *sir* ^−^, respectively. The nicked form of *HML′* circle is marked N. The profile of topoisomers in each strain was examined using NIH image software, and presented on the right. Open dots denote the centers of distribution of topoisomers in the samples.

We replaced the *HML-I* silencer at *HML* with the *HMR-E* silencer or its ORC-, Abf1- or Rap1-BS in a strain designed for measuring the supercoiling of *HML* DNA (Fig. 2A, top). In this strain, a BstBI restriction fragment containing the promoters and part of the coding regions of the *HMLα* genes was replaced by a sequence from the bacterial *lacZ* gene (designated β2) [8]. The modified *HML* locus (*HML′*) excluding the silencers was bracketed by two copies of FRT (Flp1 recombination target), recognition sites for the site-specific recombinase Flp1 (Fig. 2A, top). Induction by galactose of a P_GAL_ *-FLP1* fusion gene resident elsewhere in the genome would cause recombination between the FRTs resulting in the excision of *HML′* as a minichromosome circle (Fig. 2C). Upon deproteinization, the supercoiling of the DNA circle can be examined. In addition, this strain also bears a *URA3* gene to the right of *HML′* (Fig. 2A, top). Note that silencing by *HMR-E* is directional: robust silencing exists on its Abf1 side but not its ORC side [27], [32]. As *HMR-E* is oriented away from *HML′* in strain 17 (Fig. 2A), it would promote *URA3* sielncing as a silencer, and contribute to heterochromatin within *HML′* mainly in the capacity of a protosilencer. Therefore, the set of strains shown on the left of Fig. 2A allow for the examination of both the abilities of *HMR-E* or its protosilencer constituents to silence the *URA3* gene to the right of *HML′* and to cooperate with *HML-E* silencer to establish heterochromatin structure within *HML′*.

We found that *HMR-E* in place of *HML-I* silenced *URA3*, but the ORC- Abf1- or Rap1-BS did not (Fig. 2A, right, note the minimum growth on –Ura medium and robust growth on FOA medium of strain 17, and robust growth on –Ura medium and lack of growth on FOA medium of strains 18 through 21). This confirms that *HMR-E* as a silencer can initiate silencing, whereas the ORC-, Abf1- or Rap1-BS as a protosilencer cannot.

We also examined the abilities of *HMR-E* and its protosilencer components to collaborate with *HML-E* to promote silencing within *HML′* in strains 17n through 21n that were similar with strains 17 through 21, but had *URA3* placed within *HML′* (Fig. 2B, left). Robust *URA3* silencing was found in strains 17n and 18n (Fig. 2B). *URA3* silencing also existed in strain 19n albeit to a lesser extent than that in strains 17n and 18n (Fig. 2B). On the other hand, *URA3* was not silenced in strain 20n or 21n (Fig. 2B). These results demonstrate that ORC- or Abf1-BS, but not Rap1-BS, can cooperate with *HML-E* to promote transcriptional silencing within the region they bracket. The order of the protosilencer activities of ORC-, Abf1- and Rap1-BSs is ORC-BS>Abf1-BS>Rap1-BS.

We next examined the topology of *HML′* DNA as a proxy of chromatin structure in strains 17 through 21 as well as their *sir* ^−^ (*sir3Δ*) derivatives (strain 17s through 21s, respectively). This was achieved by inducing the excision of *HML′* circles in these strains, and subjecting them to agarose gel electrophoresis in the presence of the DNA intercalator chloroquine that resolves the topoisomers of a DNA circle according to their supercoiling (Fig. 2D, left; Fig. S1). Under the electrophoresis conditions used in this work, a more negatively supercoiled topoisomer migrated more slowly in the gel. The center of distribution of all the topoisomers of a circle is an indicator of the overall supercoiling of the circle (Fig. 2D, open dots).

As shown in Fig. 2D, the topoisomers of the *HML′* circle in strain 17 migrated markedly more slowly than those from strain 17s where *HML′* chromatin was derepressed (compare 17 and 17s). Therefore, *HML′* circle from strain 17 exhibited higher negative supercoiling than that from 17s, indicating that *HMR-E* together with *HML-E* promoted the formation of heterochromatin. The topology of *HML′* DNA in strain 18s, 19s, 20s or 21s was similar with that in strain 17s (Fig. S1), demonstrating that derepressed chromatin at *HML′* was not affected by the presence of any of the silencing elements in place of *HML-I* silencer.

The negative supercoiling of *HML′* circle in strain 21 was significantly reduced compared to that in strain 17, but was slightly higher than that from strain 17s (Fig. 2D, compare 21 with 17 and 17s). This suggests that in strain 21, *HML-E* alone cannot establish fully mature heterochromatin. The supercoiling of *HML′* DNA in strain 20 was similar to that in strain 21, suggesting that the Rap1-BS does not significantly enhance the ability of *HML-E* to form heterochromatin.

The topoisomers of *HML′* circle from strain 18 consisted of two distinct portions with one migrating similarly as *HML′* circles from strain 17 (designated *SIR* ^+^) and the other as the *HML′* circles from the *sir* ^−^ strain 17s (designated *sir* ^−^) (Fig. 2D, compare 18 with 17 and 17s). A similar result was obtained for strain 19. We have previously shown that silent *HML* circles without silencers would gradually lose their high negative supercoiling and assume a topology similar to circles in *sir^−^* cells when the host cells progress in the cell cycle, suggesting that heterochromatin dissociated from silencers is subject to disruption during cell cycle progression (Fig. 2C) [8]. The *HML′* circles excised from strains 18 and 19 (as well as strains 17, 20 and 21) all lack silencers. The *sir^−^* circles in strain 18 or 19 were therefore the result of disruption of heterochromatin on *HML′* circle during the 2.5 hr galactose induction for circle excision in which cells continued to grow. The fact that *sir^−^* circles existed in strains 18 and 19 but not 17 suggests that heterochromatin formed at *HML′* in the presence of ORC- or Abf1-BS is more susceptible to disruption than that formed in the presence of *HMR-E*. In other words, heterochromatin formed by ORC- or Abf1-BS together with *HML-E* is less stable compared to that formed by *HMR-E* and *HML-E* silencers. Note that the relative abundance of *sir^−^* circles in strain 19 was detectably more than that in strain 18 (Fig. 2D, compare 19 with 18), suggesting that heterochromatin formed by Abf1-BS is moderately less stable than that formed by ORC-BS.

Taken together, results from the above analyses of *HML′* DNA topology suggest that ORC-, Abf1- and Rap1-BSs from the *HMR-E* silencer have distinct abilities to cooperate with *HML-E* to form heterochromatin structure. ORC-BS has the strongest ability, and Rap1-BS the weakest.

To complement the DNA topology-based assay of chromatin state of *HML′* in strains 17 through 21, we also mapped *HML′* chromatin with micrococcal nuclease (MNase) digestion and indirect end-labeling [36]. Results from this experiment revealed that *HML′* chromatin in strains 17 through 21 exhibits heterochromatic (SIR-dependent) characteristics to various degrees, with strain 17 having the most heterochromatic characteristics, and strains 20 and 21 having the least, and strain 18 having more heterochromatic characteristics than 19 (Fig. S2). This suggests that the order of the abilities of ORC-, Abf1- and Rap1-BSs to contribute to the fromation of heterochromatin structure is ORC-BS>Abf1-BS>Rap1-BS, which is consistent with our conclusion on the order of activities of these protosilecners based on data from analyzing gene silencing and DNA topology at *HML′* (Fig. 2B and 2D). This further validates the use of DNA supercoiling as an indicator of chromatin state.

In summary, results from our studies of gene silencing, DNA topology and primary chromatin structure at *HML′* in strains 17n to 21n and 17 to 21 demonstrate that the *HMR-E* silencer is able to cooperate with *HML-E* to form robust, stable heterochromatin, whereas the ORC- or Abf1-BS can work with *HML-E* to form a heterochromatin structure with reduced stability. On the other hand, the Rap1-BS is not able to assist *HML-E* in establishing heterochromatin. This is in contrast to the fact Rap1-BS as part of *HMR-E* makes a greater contribution to silencer function than ORC- and Abf1-BS (Fig. 1).

### Additive effects of multiple copies of Abf1-BS on the maintenance of heterochromatin

**Figure 3:**
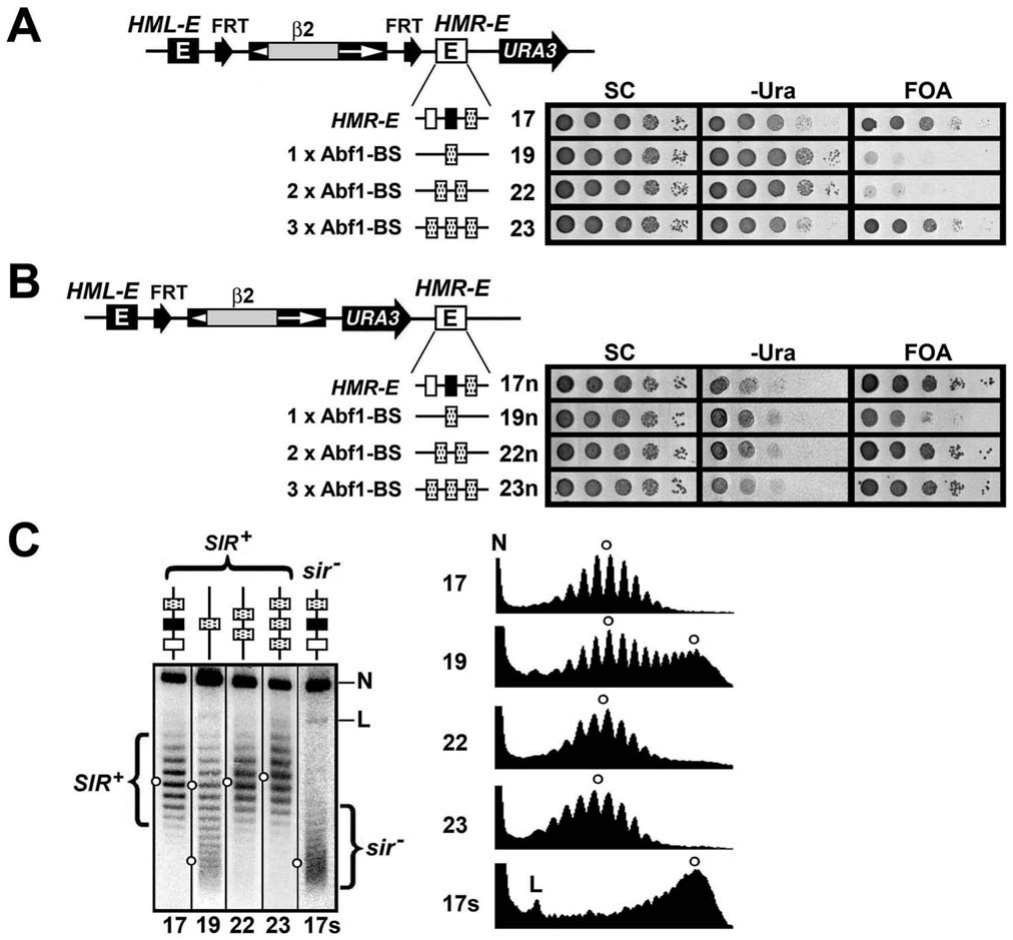
Additive effects of Abf1-BSs on the stability of heterochromatin. (A) Left, schematics of the modified *HML* locus in strains 17, 19, 22 and 23. Right, growth phenotypes. (B) Left, schematics of the modified *HML* locus in strains 17n, 19n, 22n and 23n. Right, growth phenotypes. (C) The topoisomers of *HML′* circles excised in strains 17, 19, 22, 23 and 17s were fractionated in the presence of 26 µg/ml chloroquine. The profiles of topoisomers were presented on the right.

The fact that heterochromatin formed by Abf1-BS was not as stable as that formed by *HMR-E* together with *HML-E* (Fig. 2D) prompted us to ask whether increasing the copy number of Abf1-BS could make heterochromatin more stable. To this end, we made strains 22 and 23 that were identical with 19 expect having two and three Abf1-BSs in place of *HML-I*, respectively (Fig. 3A, left). *URA3* was silenced in strain 23 (Fig. 3A, growth phenotypes of 23), suggesting that three tandem Abf1-BSs in the context of *HML-I* have silencing function similar to *HMR-E* (Fig. 3A, compare 23 with 17). However, *URA3* silencing in strain 23, but not in strain 17, was lost when the *HML-E* silencer was deleted (data not shown). Therefore, *URA3* silencing by three tandem Abf1-BSs is dependent on *HML-E*, whereas that by *HMR-E* is not, indicating that the Abf1-BSs are not a *bona fide* silencer like *HMR-E*, but are a protosilencer with enhanced activity that is sufficient to cooperate with *HML-E* to silence *URA3* in strain 23.

To examine the effect of increasing the copy number of Abf1-BS on silencing within the *HML′* locus, we made strains 22n and 23n that were simialr with 22 and 23, respectively, but had *URA3* placed within *HML′* (Fig. 3B, left). *URA3* silencing in strains 22n or 23n was significantly higher than that in strain 19n (Fig. 3B), indiacting that increasing the copy number of Abf1-BS enhances transcriptional silencing within *HML′.*

The *HML′* circle excided from strain 22 or 23 lacked *sir^−^* topoisomers, which was similar with *HML′* circle from strain 17 (Fig. 3C, compare 22 and 23 with 17). Therefore, compared with *HML′* heterochromatin in strain 19, heterochromatin in strain 22 or 23 is more stable. This result suggests that multiple Abf1-BSs have additive effects on the stability of heterochromatin structure, which correlates with the additive effects of Abf1-BSs on transcriptional silencing.

### Protosilencers ORC-BS and Abf1-BS from *HMR-E* do not facilitate heterochromatin formation by modulating chromatin structure in preparation for SIR complex spreading

How protosilencers act to assist the formation of heterochromatin has been speculated before, but direct experimental tests of the models are lacking [24], [26], [27]. We have shown previously that the structure of chromatin in the path of SIR complex spreading affects the formation of heterochromatin [23], [32]. It is possible that a protosilencer serves to modulate chromatin prior to heterochromatin formation in a way that favors the spread of SIR complex. This model is consistent with the fact that the association of ORC, Abf1 or Rap1 with DNA often influences the positioning of nucleosomes [23], [29]–[33].

**Figure 4:**
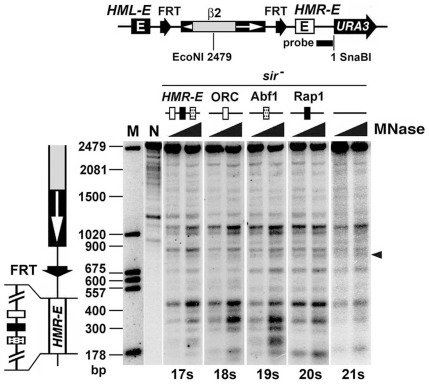
Protosilencers do not modulate chromatin structure in the absence of Sir proteins. Top, the modified *HML* locus in strain 17s. The black bar indicates the sequence corresponding to the probe used in indirect end labeling. Bottom, chromatin mapping in strains 17s through 21s by MNase digestion and indirect end labeling. MNase treated chromatin in each strain was digested with SnaBI and EcoNI and fractionated on an agarose gel. After Southern-blotting, DNA fragments ending at the SnaBI site were detected by hybridization with the probe shown at the top. The positions of the *HMR-E* silencer and FRT site are shown on the left of the blot. M, DNA markers. N, naked genomic DNA from strain 17s treated with MNase.

The above hypothesis implies that ORC-, Abf1- and Rap1-BSs as protosilencers affect chromatin before (or in the absence of) the association of SIR complex with chromatin. To test this prediction, we examined chromatin at the *HML′* locus in strains 17s through 21s that are the *sir3Δ* derivatives of strains 17 through 21, respectively (Fig. 2A, left). The primary chromatin structure was mapped by MNase digestion and indirect end labeling. Chromatin in each strain was subjected to limited MNase digestion, and the DNAs from the chromatin fragments were then isolated and digested with SnaBI restriction enzyme at a site 200 bp to the right of the *HMR-E* silencer or protosilencer and EcoNI within *HML*′ (Fig. 4, top). The DNA fragments were then fractionated, and those ending at the SnaBI site to the right of *HML′* were detected with a probe corresponding to a 200 bp sequence indicated by a bar at the top of Fig. 4.

As shown in Fig. 4, the profiles of MNase cleavage at *HML′* in strains 17s though 20s were not significantly different from each other, or from that of 21s (compare 17s through 20s with 21s), despite the existence of some subtle differences, such as the slight reduction in MNase sensitivity of a site marked by an arrowhead in strain 18s compared with that in the other strains (Fig. 4). This is consistent with the fact that *HML′* DNA in strains 17s through 21s assumed a similar topology (Fig. S1). Therefore, the presence of protosilencer ORC-BS, Abf1-BS or Rap1-BS did not affect the overall structure of derepressed chromatin, which argues against the idea that a protosilencer helps rearrange chromatin in preparation for SIR complex spreading.

### *ARS1* can counteract cell cycle-dependent disruption of heterochromatin

**Figure 5:**
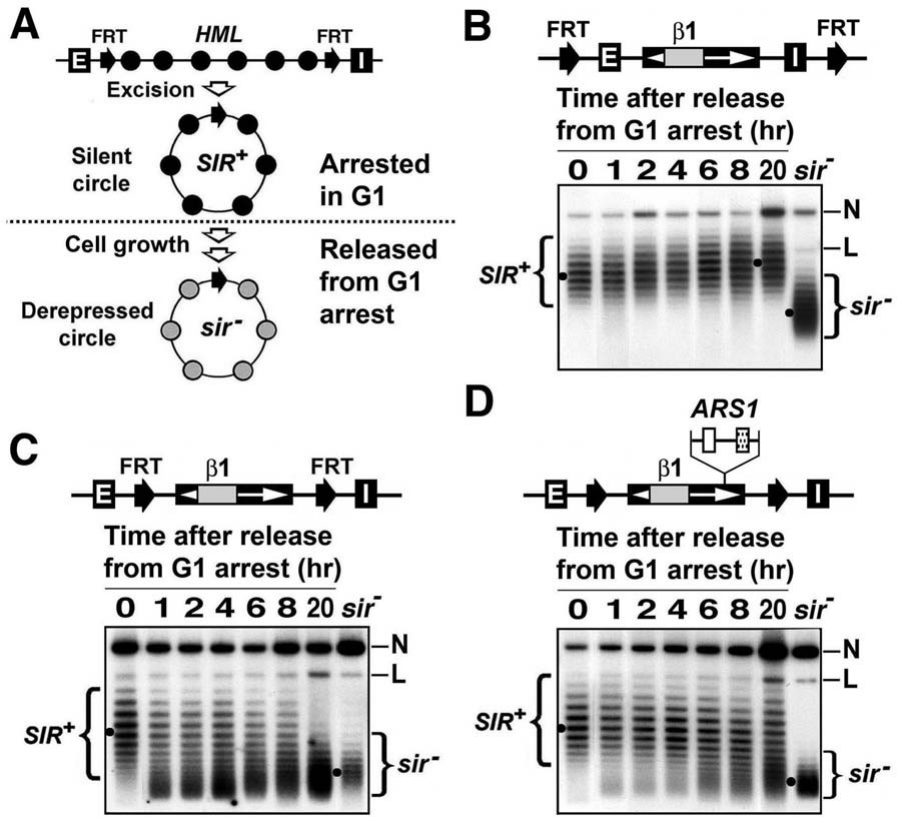
*ARS1* placed at *HML* can partially offset the disruption of heterochromatin during cell growth. (A) Method for examining the stability of heterochromatin on *HML* circle. This is identical to the method shown in Fig. 2C except that *HML* circle is excised from cells arrested in G1 phase by α-factor treatment, which avoids disruption of heterochromatin on the *HML* circle during the 2.5 hr galactose treatment for circle excision. Cells were then shifted to fresh YPD medium (1% yeast extract +2% bacto-peptone +2% dextrose) without α-factor, and allowed to grow. Heterochromatin on *HML* circle lacking silencers is subject to disruption during cell growth. Filled and shaded circles denote nucleosomes in silent and derepressed chromatins, respectively. (B, C and D) Effects of cell growth on heterochromatin on *HML′* circles in strain YXB10 (B), YXB5 (C) and strain 24 (D). All these strains have a BstBI restriction fragment containing the promoters and part of the coding regions of the *HMLα* genes replaced by a sequence from the bacterial *lacZ* gene (designated β1) [8]. Cells of each strain grown in YPR were first arrested in G1 by a 2.5 hr α-factor treatment, followed by a 2.5 hr 2% galactose treatment to excise the *HML′* circle. Cells were then shifted and diluted into fresh YPD without α-factor and further incubated for 20 hr. Aliquots of the culture were taken after 0, 1, 2, 4, 6, 8, and 20 hr. DNA was isolated and fractionated by agarose gel electrophoresis in the presence of 17 µg/ml chloroquine. N and L, nicked and linear forms of the *HML′* circle, respectively. Topoisomers corresponding to the heterochromatic and derepressed states of *HML′* circles are designated *SIR* ^+^ and *sir^−^*, respectively.

The fact that all the silencers flanking the *HML* and *HMR* loci are composed of two or three binding sites for ORC, Abf1 and Rap1 raises the question of whether other naturally occurring combinations of these sites could also promote the formation of heterochromatin. The autonomous replicating sequence 1 (*ARS1*) contains an ORC-BS (also named ACS, *ARS* consensus sequence) and an Abf1-BS. *ARS1* located on chromosome IV is a well-studied replication origin that fires early in S phase. We investigated if *ARS1* could act to maintain heterochromatin when ectopically placed at the *HML* locus. *HML* circle containing silencers maintains its silenced state (reflected by its characteristically high negative superhelical density) indefinitely during cell cycle progression of the host, whereas *HML* circle lacking silencers gradually loses its silent state and assumes a depressed state (Fig. 5A) [8]. We tested whether *HML* circle containing *ARS1* instead of its endogenous silencers could maintain its silenced state during cell cycle progression.

Strain YXB10 has two FRTs flanking *HML′* including the *HML-E* and *–I* silencers (Fig. 5B, top), whereas strain YXB5 has FRTs flanking *HML′* excluding the silencers (Fig. 5C, top). Each strain has a BstBI restriction fragment containing the promoters and part of the coding regions of the *HMLα* genes replaced by a sequence from the bacterial *lacZ* gene (designated β1) [8]. *ARS1* was inserted in the middle of *HML′* in strain YXB5 to make strain YXB125 (Fig. 5D, top). Cells of each strain were first arrested in G1 phase by α-factor treatment. The *HML′* circle was then excised. Since the host cells were in G1 and not progressing in the cell cycle, the *HML′* circle in each strain was stably maintained regardless of whether the silencers are present (Fig. 5B though 5D, lanes 0). The cells were then released from G1 arrest and allowed to grow in fresh YPD medium (without α-factor), and the topology of the *HML′* circle was measured at a series of time points afterwards. Consistent with our earlier findings, *HML′* circle containing the *E* and *I* silencers remained highly negatively supercoiled throughout the 20 hr of cells growth (Fig. 5B, compare lane 20 with lane 0), whereas *HML′* circle lacking silencers gradually lost its high negative supercoiling (Fig. 5C, compare lanes 1, 2, 4, 6, 8, and 20 with lanes 0 and *sir* ^−^). Regarding *HML′* circles bearing *ARS1* but not the *E* and *I* silencers, only a portion of them lost their high negative supercoiling, the rest retained their original topology during the 20 hours of cell growth (Fig. 5D, compare lane 20 with lane 0). Therefore, *ARS1* is able to counteract, or slow down, cell cycle-dependent disruption of heterochromatin structure.

### *ARS1* can promote the formation of a putative partially heterochromatic structure in a S-phase dependent manner

How does *ARS1* antagonize disruption of heterochromatin? One possibility is that it repairs damaged/euchromatinized part of heterochromatin by promoting local *de novo* formation of heterochromatin. This hypothesis is reasonable since *ARS1* contains an ORC-BS and an Abf1-BS that may cooperate to recruit Sir proteins, especially when it is placed at the *HML* locus that is in a context (close to telomere III-L) believed to be conducive for heterochromatin formation [47], [48]. To test this idea, we examined whether derepressed circular *HML′* minichromosome lacking silencers but bearing *ARS1* was able to form SIR-dependent chromatin upon activation of *sir3-8*, a conditional allele of *SIR3*.

**Figure 6:**
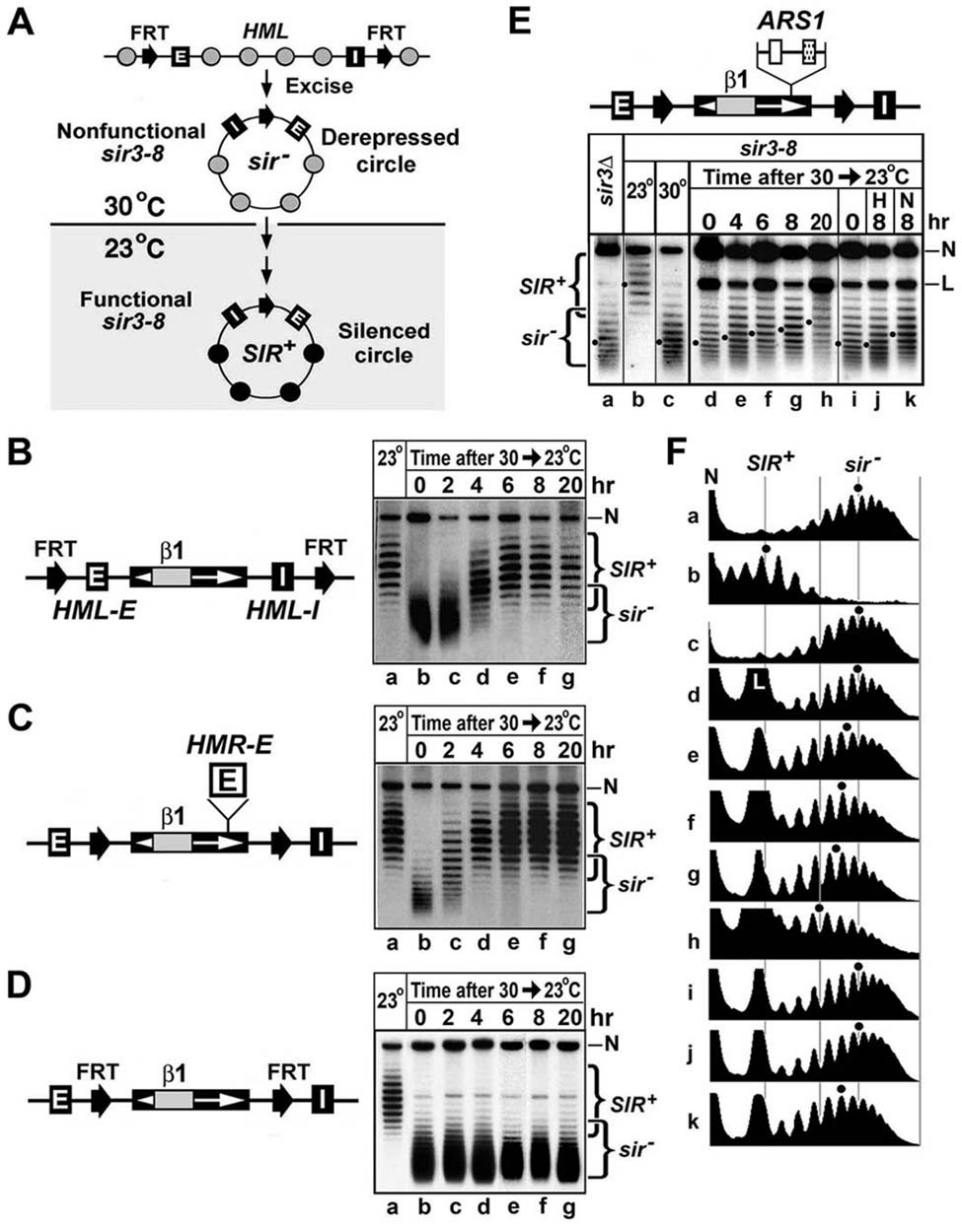
*ARS1* can promote the establishment of a *SIR*-dependent chromatin on extra-chromosomal circles in a S-phase dependent fashion. (A) Method for investigating the *de novo* establishment of heterochromatin on *HML* circle. The *HML* locus including the *E* and *I* silencers is flanked by two FRTs in a *sir3-8* strain. *HML* circle is excised in cells grown at 30°C. Cells are then shifted to fresh YPD medium and grown further at 23°C, which activates *sir3-8* and allows the formation of heterochromatin on the *HML* circle. Shaded and filled circles represent nucleosomes in derepressed chromatin and heterochromatin, respectively. Cells of strain YXB141 (B), strain 25 (C) or strain 26 (D) were grown to log phase and then further incubated for 2.5 hr in the presence of 2% galactose. Cells were pelleted and resuspended in fresh YPD medium, and were incubated for 20 hr at 23°C. Samples were taken for DNA isolation at the indicated time points. DNA isolated from cells was fractionated by agarose gel electrophoresis in the presence of 17 µg/ml chloroquine. (E) Top, modified *HML* allele in strain 27. The *ARS1* sequence inserted at *HML′* is indicated. Cells of strain 27 were initially grown at 30°C. An aliquot of the culture was incubated for 2.5 hr in the presence of 2% galactose to induce the excision of the *HML′* circle. Cells were then pelleted and resuspended in fresh YPD medium and further grown for 20 hr at 23°C. Samples were taken for DNA isolation after the indicated times (samples d through h). Another aliquot was treated with α-factor for 2.5 hr at 30°C to arrest cells in G1 phase of the cell cycle. Galactose was then added to this culture that was incubated at 30°C for another 2.5 hr. A third of this culture was used to isolate DNA (sample i). The rest was shifted to fresh YPD medium, and half of it was grown in the presence of 0.2 M HU (sample j) and the other in the presence of 20 µg/ml nocodazole (sample k), for 8 hr at 23°C. DNA isolated from each sample of cells was fractionated by agarose gel electrophoresis in the presence of 17 µg/ml of chloroquine. Dots indicate the Gaussian center of the topoisomer distribution of *HML*′ circles. (F) The distribution of topoisomers in each sample examined in (E) was determined using the NIH image software. The centers of distribution are marked by dots. Note that all the strains examined here have a BstBI restriction fragment containing the promoters and part of the coding regions of the *HMLα* genes replaced by a sequence from the bacterial *lacZ* gene (designated β1) [8].

The temperature-sensitive *sir3-8* allele is functional at 23°C but not at 30°C [49]. We have made a *sir3-8* strain bearing FRTs flanking *HML* including the *HML-E* and *–I* silencers (Fig. 6A, top), and shown that chromatin on *HML* circle excised at 30°C was converted from derepressed (*sir* ^−^) state to silenced (*SIR* ^+^) state after the growth temperature was shifted to 23°C as illustrated in Fig. 6A [50]. The state of chromatin in such experiments was followed by measuring the negative supercoiling of the *HML* circle.

As expected, the *HML′* circle bearing silencers excised in the *sir3-8* strain YXB141 (Fig. 6B, left) had high negative supercoiling at 23°C, and reduced negative supercoiling at 30°C (Fig. 6B, right, lanes a and b), confirming the existence of heterochromatin at 23°C and euchromatin at 30°C on the circle. Importantly, after cells containing derepressed (*sir* ^−^) *HML′* circle were shifted from 30°C to 23°C and allowed to grow further, the negative supercoiling of the circle increased and reached the level of a *SIR* ^+^ circle by hour 6 (Fig. 6B, compare lanes b through g with a). This confirms the conversion of derepressed chromatin on the *HML′* circle to heterochromatin after the activation of *sir3-8*. A similar result was obtained with an *HML′* circle bearing the *HMR-E* silencer instead of the *HML-E* and *–I* silencers (Fig. 6C). On the other hand, derepressed (*sir* ^−^) *HML′* circle lacking silencers was not converted to silent (*SIR* ^+^) circle upon activation of *sir3-8* (Fig. 6D). Taken together, the above results demonstrate that *HML* and *HMR* silencers on an *HML′* circle can promote efficient *de novo* establishment of heterochromatin.

To test if *ARS1* could promote heterochromatin formation, we inserted it within the *HML′* locus of strain 26 (Fig. 6D, left) to make strain 27 (Fig. 6E, top). As expected, *HML′* circle excised from strain 27 grown at 23°C had high negative supercoiling, whereas that at 30°C had lower negative supercoiling (Fig. 6E, lanes b and c), confirming that heterochromatin was formed on the *HML′* circle at 23°C but not at 30°C. We then examined if derepressed *HML′* circle preexistent in strain 27 could be converted to silenced state after the growth temperature was changed from 30°C to 23°C. As shown in Fig. 6E and 6F, *HML′* circles examined at hours 4, 6, 8 and 20 were more negatively supercoiled than the starting sample (hour 0) by 1, 1.5, 2 and 3 negative supercoils, respectively (compare e, f, g and h with d). However, these increases in negative supercoiling (3 or less negative supercoils) were significantly smaller than the difference of 6.5 negative supercoils between silent and derepressed states of *HML′* circle (Fig. 6E and 6F, compare b and c). These results are consistent with the notion that *ARS1* promotes the formation of a partially silenced, or intermediate, chromatin structure on the *HML′* circle.

The *de novo* establishment of heterochromatin at the *HM* loci has been previously shown to depend on passage of the host cell through S phase of the cell cycle, but not DNA replication *per se* [49]–[52]. We tested if the formation of the putative intermediate chromatin structure on *ARS1*-contining *HML′* circle was also S-phase dependent. We first excised the *HML′* circle in strain 27 cells that were arrested in G1 (by α-factor) at 30°C, and then shifted the cells to 23°C and allowed them to grow for 8 hours in the presence of either hydroxyurea (HU) that arrests cells in early S-phase, or nocodazole that arrests cells in G2/M phase. During the 8 hr incubation in the presence of HU, cells were able to progress from G1 (point of α-factor arrest) to early S phase (point of HU arrest). The topology of *HML′* DNA in these cells was not significantly different from that in cells before the incubation (Fig. 6E and 6F, compare j with i). Therefore, blocking cells from progressing beyond early S phase eliminates the *SIR3*-dependent change in chromatin structure on the *HML′* circle containing *ARS1*. On the other hand, during the 8 hr incubation in the presence of nocodazole, cells progressed from G1 to G2/M (point of nocodazole arrest) of the cells cycle. The *HML′* circle in these cells was 1.5 supercoils more negatively supercoiled than that in cells before the incubation (Fig. 6E and 6F, compare k with i). Therefore, cell cycle progression from G1 to G2/M induces a *SIR3*-dependent change in chromatin structure on *HML′* circle containing *ARS1*. These results suggest that the putative role of *ARS1* in promoting the formation of a SIR-dependent chromatin conformation requires the host to traverse through S phase of the cell cycle.

## Discussion

Transcriptional silencing is a conserved mechanism of region-specific gene repression that may affect large regions of the genome. The locus-specificity of silencing in yeast is determined by *cis*-acting silencers and telomeres that serve to initiate the formation of a repressive heterochromatin structure. Silencers each consist of two or three of ORC-BS, Abf1-BS and Rap1-BS and serve as a recruitment center for the SIR complex. Individual ORC-BS, Abf1-BS and Rap1-BS do not have the ability to initiate *de novo* formation of heterochromatin, but can facilitate silencing by a *bona fide* silencer at a distance, and are called protosilencers. However, intriguingly, ORC-BSs also exist at all replication origins, or autonomous replication sequences (ARSs), and bind ORC involved in the initiation of DNA replication. Abf1-BSs and Rap1-BSs are also found at many gene promoter regions and associate with Abf1 and Rap1, respectively, as general regulatory factors involved in gene activation [53]–[55]. Therefore, whether these binding sites function in silencing, replication initiation, or gene activation is likely dependent on the genomic environment. Moreover, how efficiently a silencer or protosilencer functions also depends on its context [27].

The *HMR-E* silencer is the strongest among all the silencers in promoting transcriptional silencing [27]. Of the ORC-BS, Abf1-BS and Rap1-BS components of *HMR-E*, we found Rap1-BS to be especially important for its function (Fig. 1). A recent analysis of a synthetic minimum *HML-E* silencer consisting of an ORC-BS, a Rap1-BS and a Sum1-BS also suggests that Rap1-BS plays a more important role than the other two elements [56]. Both ORC and Rap1 are believed to contribute to silencer function by recruiting Sir3 and/or Sir4 proteins [1]. Rap1 dierctly binds Sir3 and Sir4, whereas ORC binds Sir1 which in turn binds Sir4. How Abf1 participates in the initiation of silencing has not been resolved, although there has been anecdotal information that Abf1 interacts with Sir3. Why the Rap1-BS is particularly important for *HMR-E* function is not clear. One possibility is that because Rap1-BS is located in the middle of *HMR-E* (flanked by ORC- and Abf1-BSs) (Fig. 1A), it is critical for the cooperation of the three silencer binding proteins. Loss of Rap1-BS may severely hinder the collaboration between ORC and Abf1 in recruiting SIR complex, due to the relatively large distance between ORC-BS and Abf1-BS. On the other hand, loss of Abf1-BS may not affect the cooperation between ORC and Rap1, and loss of ORC-BS may not affect the cooperation between Rap1 and Abf1.

Transcriptional silencing is subject to regulation by many factors including *SAS2* encoding a histone H4 acetyltransferase. *SAS2* plays a positive role in silencing as *sas2Δ* reduces *HM* silencing as well as telomeric silencing [40]–[43]. Consistently, we showed in this report that silencing of *TRP1* by intact *HMR-E*, or *HMR-E* lacking ORC-BS or Abf1-BS is reduced by *sas2Δ* (Fig. 1). Given that the SIR complex preferentially binds deacetylated nucleosomes, acetylation of histone H4-K16 by Sas2 in euchromatin has been proposed to hinder ectopic spreading of SIR complex from heterochromatin, thereby helping restricting SIR complexes to silent loci [57], [58]. Global reduction in H4-K16 acetylation as a result of *sas2Δ* may allow a subset of Sir proteins to leave *HM* loci and associate with euchromatin regions, thereby reducing *HM* silencing. Consistent with this model, we have shown that in *sas2Δ* cells *HML* heterochromatin adopts an intermediate state between fully silent and derepressed structures [43]. As we have also shown that *sas2Δ* and *orc5-1* have a synthetic effect on *HMR* silencing, we envisioned that Sas2 might regulate silencing by affecting ORC function at the *HMR-E* silencer [43]. However, the positive role of Sas2 in *HMR-E* silencing does not seem to depend on the presence ORC-BS in the silencer (Fig. 1). Therefore, it is unlikely that Sas2 contributes to *HMR-E* function *via* regulating ORC.

Intriguingly, *sas2Δ* enhances silencing by *HMR-E* deleted for Rap1-BS (*HMR-EΔR*) (Fig. 1). In other words, Sas2 plays an inhibitory role in the function of *HMR-EΔR*. It seems that Rap1-BS helps determine the mode (positive vs. negative) of function of Sas2 in silencing by *HMR-E*. Given that *HMR-E* function is affected by its chromatin context [32], it is possible that *sas2Δ* induced reduction in H4-K16 acetylation affects chromatin around *HMR-E* in a manner that is conducive to *HMR-EΔR*, but inhibitory to *HMR-E*, *HMR-EΔO* and *HMR-EΔA*. Rap1 interacts with Rif1 and Rif2 proteins, in addition to the SIR complex, and Rif1 and Rif2 are required for full silencing at *HMR* [59], [60]. It would be interesting to explore whether Rif1 and/or Rif2 are involved in determing the regulation of *HMR-E′* by Sas2.

It is interesting that although Rap1-BS plays a larger and unique role in *HMR-E* silencer function compared to ORC-BS and Abf1-BS, it does not serve as a protosilencer for *HML-E* as does ORC-BS or Abf1-BS (Fig. 2). Therefore, the functions of Rap1-BS as part of *HMR-E* silencer and as a protosilencer may be mechanistically different. It is noteworthy that the Rap1-BS in *HMR-E* (5′-AAACCCATCAACC-3′) is a variant of a consensus sequence (5′-ACACCCRYACAYM-3′; M, A or C; R, A or G; Y, C or T) for Rap1 recognition [55], [61]. Other Rap1-BSs existing elsewhere in the genome are distinct variants of the consensus. Since Rap1-BSs exhibit considerable sequence heterogeneity, they are likely to have different affinities for Rap1, which may affect their functions [55], [61], [62]. This may be the reason why unlike Rap1-BS from *HMR-E*, the Rap1-BS from *HML-E* silencer and UASα (a Rap1-BS) both exhibit protosilencer functions [2], [24], [27].

Given the fact that each silencer consists of a combination of two or three protosilencers, it would be reasonable to think that multiple copies of the same protosilencer should have a stronger protosilencer activity. We found this to be the case for the protosilencer Abf1-BS from *HMR-E*: three tandem Abf1-BSs display a greater activity in enhancing silencing and the stability of heterochromatin (Fig. 3). However, we have previously shown that two or three tandem Rap1-BSs could serve as a barrier to the propagation of heterochromatin instead of a stronger protosilencer [63]. It would be interesting to investigate what determines if Rap1-BSs act as protosilencers or heterochromatin barrier elements.

A protosilencer can enhance the action of a silencer or telomere located at a distance of up to several kb. The mechanism underlying this functional interaction has not been elucidated. As the establishment of silencing is mediated by the binding of SIR complexes to an array of nucleosomes, the primary chromatin structure may play a role in determining the efficiency of SIR complex association. This notion is supported by our finding that disrupting the regularity of nucleosomes by nucleosome-excluding structures blocks the spread of heterochromatin [23]. Since protosilencers ORC-BS, Abf1-BS and Rap1-BS all have the potential of modulating nucleosome positioning upon associating with their corresponding proteins [23], [29]–[33], it is possible that a protosilencer assists SIR complex propagation from a silencer by altering chromatin structure in a way that favors SIR-chromatin interaction [27]. However, we found that protosilencer ORC-BS or Abf1-BS in place of the *HML-I* silencer does not affect the primary chromatin structure between it and the *HML-E* silencer in a *sir* ^−^ background (Fig. 4), which argues against the model involving chromatin structure. An alternative hypothesis proposes that the silencer and protosilencer physically contact persistently or transiently to establish a stronger silencing center that can better recruit the SIR complex [26]. However, there has not been direct evidence supporting such a physical interaction model.

ORC-BS, or ACS (*ARS* consequence sequence), is the core component of an *ARS*, as well as a silencer. ORC-BSs also exist at subtelomeric regions where they act as protosilencers aiding in telomeric silencing [25], [64]. A single ORC-BS in place of the *HML-E* or *HML-I* silencer also acts as a protosilencer to enhance the function of the other *HML* silencer [24] (Fig. 2). *ARS1* contains an ORC-BS and an Abf1-BS required for replication origin function. Under a special circumstance (high copy expression of *FKH1*) *ARS1* in place of *HMR-E* silencer has been shown to mediate *HMR* silencing (together with *HMR-I* silencer) [65]. In this report, we showed that *ARS1* inserted at *HML* locus makes *HML* heterochromatin more resistant to cell cycle-dependent disruption (Fig. 5). Importantly, we obtained evidence suggesting that *ARS1* on an *HML* circle lacking silencers has the ability to promote the transformation of derepressed chromatin structure into an intermediate or altered structure that is between heterochromatin and derepressed chromatin (Fig. 6). Moreover, such a transformation is dependent on S-phase progression of the host, which is similar to the S-phase (but not DNA replication) requirement for *de novo* formation of heterochromatin mediated by *bona fide* silencers [49]–[52]. Based on these results, it is possible that *ARS1* repairs damages to heterochromatin (e.g., partial loss of SIR complex association) inflicted by cell cycle progression by promoting *de novo* formation of heterochromatin in limited regions.

## Supporting Information

Figure S1 **Protosilencers ORC-BS, Abf1-BS and Rap1-BS do not affect derepressed** ***HML*** **chromatin.** Cells of each of the strains 17s through 21s were grown in YPR to late log phase, and were then treated with 2% galactose for 2.5 hr. Nucleic acids were isolated and fractionated in the presence of 26 µg/ml chloroquine. The topoisomers were labeled *sir* ^−^. The relevant silencing element in each strain is shown at the top. The nicked and linear forms of *HML′* circle are marked N and L, respectively.
(TIF)

Figure S2 **Contributions of protosilencers to heterochromatin structure.** Top, the modified *HML* locus in strains 17 and 17s. The black bar indicates the sequence corresponding to the probe used in indirect end labeling. Bottom, chromatin mapping in strains 17 through 21, as well as 17s by MNase digestion and indirect end labeling. MNase treated chromatin in each strain was digested with SnaBI and EcoNI and fractionated on an agarose gel. After Southern-blotting, DNA fragments ending at the SnaBI site were detected by hybridization with the probe shown at the top. The positions of the *HMR-E* silencer and FRT site are shown on the left of the blot. M, DNA markers. N, naked genomic DNA from strain 17s treated with MNase. The profile of MNase cleavage at *HML′* in strain 17 (*SIR* ^+^) was clearly distinct from that in 17s (*sir* ^−^) (note the strain 17-specifc bands indicated by diamonds and 17s-specific bands labeled by filled cricles), which is consistent with the marked difference in *HML* DNA topology between strains 17 and 17s (Fig. 2D). This confirms the formation of heterochromatin at *HML′* in strain 17 with a primary structure different from derepressed chromatin in strain 17s. As shown in Fig. 4, MNase digestion pattern in strains 18s to 21s was not significantly different from that in strain 17s, suggesting that the presence of protosilencer ORC-BS, Abf1-BS or Rap1-BS did not affect the overall structure of derepressed chromatin at *HML′*. As such, *HML′* chromatin in strain 17s can represent derepressed *HML′* chromatin in strains 18s to 21s. MNase digestion pattern in strain 21 shares several characteristics with that of 17s (bands indicated by filled circles in both lanes 21 and 17s), and also share some features with that of strain 17 (bands indicated by diamonds in lane 21). Therefore, *HML′* chromatin in strain 21 has features of both derepressed chromatin (as in strain 17s) and heterochromatin (as in strain 17). In addition, there were two MNase sensitive sites (indicated by open circles) that existed only in strain 21. These results support the notion that an intermediate chromatin structure different from both heterochromatin and derepressed chromatin is formed in strain 21 by the *HML-E* silencer alone. This notion was also supported by the fact that the negative supercoiling of *HML′* DNA in strain 21 was lower than that in strain 17, but higher than that in strain 17s (Fig. 2D). Strain 20 was identical with 21 regarding MNase digestion of *HML′* chromatin, which is in line with the fact these two strains were also identical with respect to the supercoiling of *HML* DNA (Fig. 2D). This further demonstrates the inability of the Rap1p site in strain 20 to assist *HML-E* silencer in establishing mature heterochromatin. MNase digestion at *HML′* in strain 18 was similar but not identical with that of strain 17 (note 18 and 17 share bands indicated by diamonds, but 18 has an extra band denoted by an open dot). This suggests that heterochromatin formed at *HML* in strain 18 has a conformation that is similar, but not identical, with that in strain 17. Compared with strain 18, strain 19 lost a heterochromatin-specific MNase site denoted by a diamond), and gained two derepressed chromatin-specific sites (denoted by filled dots) at *HML′*. Taken together, the above results demonstrate that *HML′* in strains 17 though 20 exhibit less and less heterochromatic features, and more and more derepressed chromatin-specific features. This suggests that the ORC-, Abf1- and Rap1-BSs from the *HMR-E* silencer have distinct abilities to contribute to the structure of heterochromatin, with the order of their activities being ORC-BS>Abf1-BS>Rap1-BS.
(TIF)

Table S1 **Yeast strains.**
(XLSX)
