# Supplementary figures and images for: Functions of Protosilencers in the Formation and Maintenance of Heterochromatin in Saccharomyces cerevisiae
Source: PLoS One. 2012 May 17;7(5):e37092. doi: 10.1371/journal.pone.0037092 (PMC3355138; doi:10.1371/journal.pone.0037092)

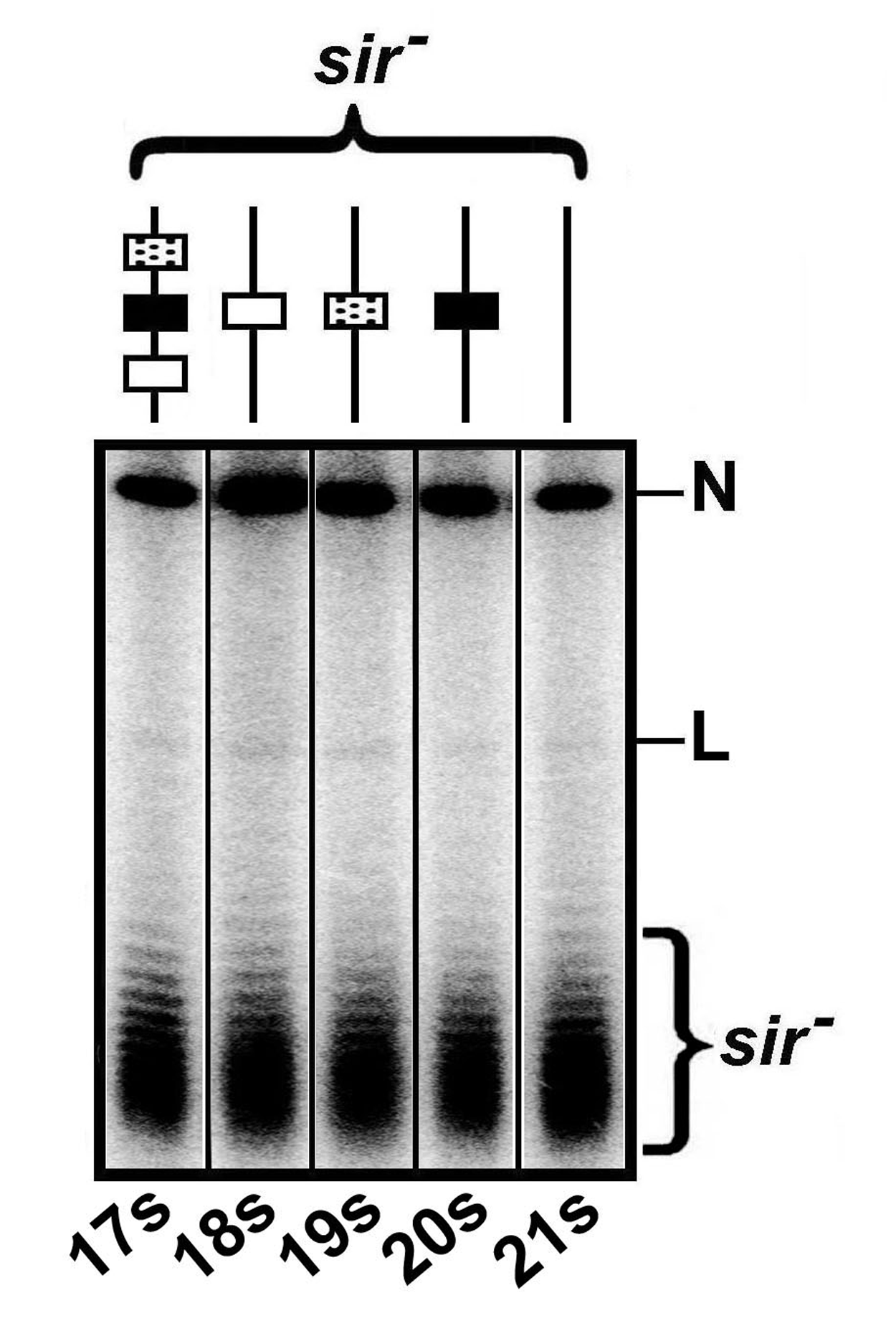

Supplement: Figure S1 — Protosilencers ORC-BS, Abf1-BS and Rap1-BS do not affect derepressed HML chromatin. Cells of each of the strains 17s through 21s were grown in YPR to late log phase, and were then treated with 2% galactose for 2.5 hr. Nucleic acids were isolated and fractionated in the presence of 26 µg/ml chloroquine. The topoisomers were labeled sir −. The relevant silencing element in each strain is shown at the top. The nicked and linear forms of HML′ circle are marked N and L, respectively. (TIF) [file pone.0037092.s001.tif]

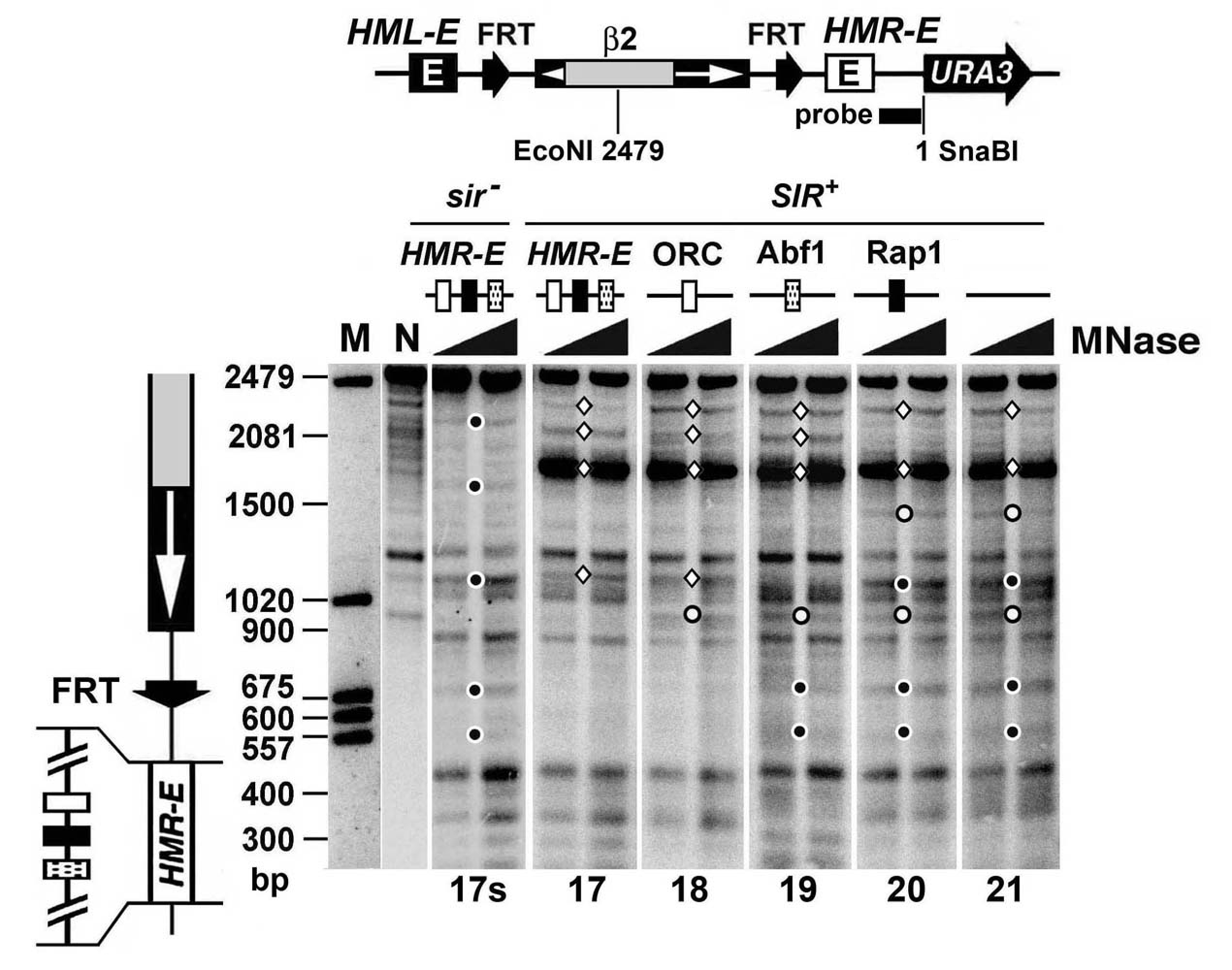

Supplement: Figure S2 — Contributions of protosilencers to heterochromatin structure. Top, the modified HML locus in strains 17 and 17s. The black bar indicates the sequence corresponding to the probe used in indirect end labeling. Bottom, chromatin mapping in strains 17 through 21, as well as 17s by MNase digestion and indirect end labeling. MNase treated chromatin in each strain was digested with SnaBI and EcoNI and fractionated on an agarose gel. After Southern-blotting, DNA fragments ending at the SnaBI site were detected by hybridization with the probe shown at the top. The positions of the HMR-E silencer and FRT site are shown on the left of the blot. M, DNA markers. N, naked genomic DNA from strain 17s treated with MNase. The profile of MNase cleavage at HML′ in strain 17 (SIR +) was clearly distinct from that in 17s (sir −) (note the strain 17-specifc bands indicated by diamonds and 17s-specific bands labeled by filled cricles), which is consistent with the marked difference in HML DNA topology between strains 17 and 17s (Fig. 2D). This confirms the formation of heterochromatin at HML′ in strain 17 with a primary structure different from derepressed chromatin in strain 17s. As shown in Fig. 4, MNase digestion pattern in strains 18s to 21s was not significantly different from that in strain 17s, suggesting that the presence of protosilencer ORC-BS, Abf1-BS or Rap1-BS did not affect the overall structure of derepressed chromatin at HML′. As such, HML′ chromatin in strain 17s can represent derepressed HML′ chromatin in strains 18s to 21s. MNase digestion pattern in strain 21 shares several characteristics with that of 17s (bands indicated by filled circles in both lanes 21 and 17s), and also share some features with that of strain 17 (bands indicated by diamonds in lane 21). Therefore, HML′ chromatin in strain 21 has features of both derepressed chromatin (as in strain 17s) and heterochromatin (as in strain 17). In addition, there were two MNase sensitive sites (indicated by open circ [file pone.0037092.s002.tif]
